# Morphology, Crystallization and Thermal Behaviors of PLA-Based Composites: Wonderful Effects of Hybrid GO/PEG via Dynamic Impregnating

**DOI:** 10.3390/polym9100528

**Published:** 2017-10-19

**Authors:** Shikui Jia, Demei Yu, Yan Zhu, Zhong Wang, Ligui Chen, Lei Fu

**Affiliations:** 1 School of Materials Science and Engineering, Shaanxi University of Technology, Hanzhong 723000, China; yanzhu@163.com (Y.Z.); wangzhong6110@163.com (Z.W.); harvy_chen@126.com (L.C.); slglisa@163.com (L.F.); 2 School of Science, Xi’an Jiaotong University, Xi’an 710049, China

**Keywords:** poly-(lactic acid), hybrid GO/PEG, dynamic impregnating, morphology, thermal behaviors

## Abstract

In this paper, a dynamic impregnating device, which can generate supersonic vibration with the vacuum-adsorbing field, was used to prepare the hybrid graphene oxide (GO)/polyethylene glycol (PEG). Interestingly, the hybrid GO/PEG under dynamic impregnating and/or internal mixing was introduced into poly-(lactic acid) (PLA) matrix via melting-compounding, respectively. On one hand, compared with the internal mixing, the hybrid GO/PEG with the different component ratio using dynamic impregnation had a better dispersed morphology in the PLA matrix. On the other hand, compared with the high molecular weight (*M*_w_) of PEG, the hybrid GO/PEG with low *M*_w_ of PEG had better an exfoliated morphology and significantly improved the heat distortion temperature (HDT) of the PLA matrix. Binding energies results indicate that low *M*_w_ of PEG with GO has excellent compatibility. Dispersed morphologies of the hybrid GO/PEG show that the dynamic impregnating had stronger blending capacity than the internal mixing and obviously improved the exfoliated morphology of GO in the PLA. Crystallization behaviors indicate that the hybrid GO/PEG with the low *M*_w_ of PEG based on dynamic impregnating effectively enhanced the crystallinity of PLA, and the cold crystallization character of PLA disappeared in the melting process. Moreover, the storage modulus and loss factor of the PLA-based composites were also investigated and their HDT was improved with the introduction of hybrid GO/PEG. Furthermore, a physical model for the dispersed morphology of the hybrid GO/PEG in the PLA matrix was established. Overall, the unique blending technique of hybrid GO/PEG via dynamic impregnating is an effective approach to enhance the property range of PLA and is suitable for many industrial applications.

## 1. Introduction

In recent years, the interest in biodegradable polymers has increasingly grown all over the world. However, biodegradable polymers cannot be widely used via the choke point induced by their prices, thermostability, and mechanical properties [1]. Notably, Poly-(lactic acid) (PLA) is a biodegradable polyester which has been used in a wide range of applications such as agriculture [2], medicine [3], packaging [4], etc. Except for good thermoplasticity and processability, PLA has good biodegradability, biocompatibility, and physical properties, such as high strength and modulus. However, PLA has very low crystallinity, leading to inferior thermostability problems during its long service life [5,6,7,8]. In order to solve these defects, blending with nanoscale particles is one of the commonly used methods [9,10,11]. In the basis of the large aspect ratio and excellent strength, some nanoparticles such as carbon nanotubes, layered silicate, and graphene nanoplates, have been used as fillers of PLA to manufacture composites with good thermal performance or unexpected properties [12,13,14,15,16].

Graphene has attracted much attention in recent years due to its outstanding electrical, thermal, and mechanical properties [17,18]. The graphene nanoplates material also has a high aspect ratio so that it can be ideal filler for polymer composites [19,20,21]. Unfortunately, the strong van der Waals interactions between graphene nanoplates generally produced the aggregation/flocculation, leading to the poor dispersion of graphene in polymer matrices during the process of melt mixing or solution coagulation. In order to weaken the aggregation/flocculation of graphene nanoplates in the PLA matrix, people presented a new improvement strategy for graphene nanoplates based on functionalizing graphene oxide (GO), which has improved compatibility with polymer matrices and stands out as a primary strategy to incorporate graphene sheets uniformly into PLA matrices [22,23,24]. Huang et al. [25] found that poly(vinylidene fluoride)/GO nanocomposites are prepared by one-step water-assisted mixing extrusion via injecting GO suspension into polymer melt. Liu et al. [26] revealed that graphene nanoplates also have been successfully modified by small molecules or biomolecules via physical interactions to obtain functional composites. Fu et al. [27] fabricated PVA/graphene nanocomposite by using nanocellulose to assist the dispersion of graphene in water. In the previous studies, the agglomeration of GO has been effectively modified by a series of strategies, while the crystallization rate of PLA phase in the GO/PLA composite was not improved significantly.

Polyethylene glycol (PEG) is a typical low molecules polymer. It is recognized as the most studied promoter for PLA, manifesting the desirable compatibility with PLA even at the loading of 30 wt %. The enhancement of the growth rate of PLA spherulites by the presence of PEG is well documented [21]. Naturally, Li et al. [28] found that the incorporation between the nucleation agent and promoter may dramatically improve the crystallization kinetics of the polymer matrix. A similar phenomenon was found in the ternary PLA system with GO and PEG [29]. It was indicated that GO and PEG, respectively, acted as the nucleation agent and the plasticizer, leading to a synergistic effect on the overall crystallization kinetics of PLA. Zhang et al. [23] used esterification between the carboxylic acid-functionalized GO and the terminal hydroxyl groups of PEG to improve the dispersion of GO in PLA, which takes advantage of the good compatibility between PEG and PLA. Actually, though the modified methods indeed exhibit great benefits to disperse GO, complex synthetic procedures and a lot of organic solvent are generally involved in the synthesis process. To simplify the fabrication process and fulfill the dispersion of GO in PLA, Qu et al. [30] designed the environmental mean for the dispersion of GO within the PLA matrix by hot water—the PEG solution, improving the dispersion of GO in PLA during the melt mixing process. However, when hot water is gradually evaporated, the GO in the directly dried GO/PEG mixture is heterogeneous and difficult to re-disperse in the PLA matrix. In other words, this method cannot be used to prepare well dispersed GO/PLA composites using PEG as a promoter. Thus, Feng et al. [31] proposed the “freezing-dried graphene/PEG masterbatch”, which can promote the dispersion of graphene and the crystallization of PLA. The dispersion level of GO in the PLA-based composite was preferably accomplished after undergoing the melt-compounding process. Importantly, the macroscopic preparation limitation of the freezing-dried graphene/PEG masterbatch will hinder its industrial application.

Vacuuming impregnation, which is an environmentally friendly process, has been frequently used to prepare the porous structure of the shape-stability phase change storage energy materials in the past years [32,33]. We have previously found that the synergy effect of vacuuming impregnation and the supersonic vibration force field could very effectively promote the dispersion of GO in molten PEG [34]. On account of the aforementioned issues such as the slow crystallization rate, complex procedures, and inferior GO dispersion within the PLA phase, in the present study we propose a simple “hybrid GO/PEG” strategy to simultaneously fulfill the good dispersion of the GO sheets in the PLA matrix and the improved crystallization ability and thermostability of PLA. We present a novel approach called dynamic impregnating treatment with vacuuming and supersonic vibration to prepare the hybrid GO/EG composite, which shows the good dispersity and absorbability within the PLA matrix during the melt-compounding process. Moreover, another main aim of this study is to examine the crystallization and thermal properties of the GO/PEG/PLA ternary composites prepared by melt-compounding via the twin-screw extruder and investigate the effects of hybrid GO/PEG under different *M*_w_ of PEG, as well as the component ratio on the final properties of PLA. The main novelty of this work is the use of the hybrid GO/PEG based on dynamic impregnating as a nucleating agent and plasticizer for improving the crystallinity and thermostability of PLA. The physical model for the dispersed morphology of the hybrid GO/PEG via supersonic vibrating and the vacuuming force field will be firstly established.

## 2. Materials and Methods

### 2.1. Materials

The PLA matrix (4032D, NatureWorks, La Vista, NE, USA) was a melt flow index of 55 g/10 min and molecular weight (*M*_w_) of 120,000 g/mol, respectively. GO (Thick: 5 nm, Diameter: 30 μm, Carbon content: 99%) was supplied by Taiji-ring nano-product Co., Ltd., Hebei, China. PEG (*M*_w_ = 4000 and/or 20,000 g/mol, Analytical reagent) was supplied by Sinopharm Chemical Reagent Beijing Co., Beijing, Ltd, Beijing, China.

### 2.2. Sample Preparation

GO were dried in a vacuum oven at 70 °C for 48 h. Then, the hybrid GO/PEG as a function of different component ratio and *M*_w_ of PEG were premixed by tumbling in a plastic zip-lock bag. Then, a novel dynamic impregnating device [35], which generates supersonic vibrating and vacuuming force field, was used to compound the GO/PEG. First, the premix was placed in a constant temperature cavity of 90 °C and melted at vacuuming condition at a pressure of 0.05 MPa for 30 min. Meanwhile, the liquid mixture of GO and PEG was vibrated by supersonic wave until the mixture was mixed homogeneously. Moreover, in order to study the effect of different processing method on dispersed morphology of hybrid GO/PEG, the hybrid GO/PEG was prepared by internal mixing at 90 °C for 30 min. Finally, all samples were dried at a constant weight in an electric vacuum oven at 30 °C for 6 h.

Prior to the blending process, the PLA was vacuum dried at 60 °C for 8 h. Afterwards, according to the formulations (shown in Table 1), PLA and a series of the hybrid GO/PEG were melt blended by an internal mixer (HAAKE RS600, HAAKE, Karlsruhe, Germany) at a rotational speed of 60 rpm and 180 °C for 15 min). All of the components were simultaneously loaded into the mixing chamber. The final composites were quickly hot-pressed at 170 °C and a pressure of nearly 5 MPa to prepare a series of flat pieces with approximately thickness of 4 mm for all measurements.

### 2.3. Characterization

Advancing contact angle measurements of two probe liquids (water and glycerol) on the GO, different *M*_w_ of PEG, and pure PLA specimens were taken using a contact angle analysis system (OCA 20LHT, data physice, Stuttgart, Germany) to evaluate the dispersive and polar surface energy ($\gamma^{d}$ and $\gamma^{p}$) of the surface free energy (SFE) of the raw materials. The error bars on the measured contact angles were determined based on the standard deviation between 5 contact angles measurements for each sample taken from all directions, including parallel and perpendicular. The final contact angle was adopted via average value of 5 samples. The detections were performed at a stabilized condition (25 °C and 55 wt % relative humidity). Relation of total SFE ($\gamma_{s}$) of solid surface to polar ($\gamma_{s}^{p}$) and dispersive ($\gamma_{s}^{d}$) surface energy is based on Equation (1) and by knowing the contact angles of a set of liquid (with determined $\gamma_{L}^{d}$ and $\gamma_{s}^{p}$) on a solid surface; $\gamma_{s}^{d}$ and $\gamma_{s}^{p}$ for the solid materials can be obtained according to Equation (2).

(1) $$\gamma_{s}=\gamma_{s}^{d}+\gamma_{s}^{p}$$

(2) $$\gamma_{L}(1+\cos\theta )=2\sqrt{\gamma_{s}^{d}+\gamma_{L}^{d}}+2\sqrt{\gamma_{s}^{p}+\gamma_{L}^{p}}$$

where s and L represent the solid and liquid surfaces, respectively, and θ is the contact angle of a liquid droplet on the solid surface. Moreover, the interfacial energy of every two raw materials can be calculated from their polar and dispersive components of surface energies on the basis of Equations (3) and (4) [36,37,38].

(3) $$\mathrm{Harmonic}\;\mathrm{mean}:\;\gamma_{12}=\gamma_{1}+\gamma_{2}-4(\frac{\gamma_{1}^{d}\gamma_{2}^{d}}{\gamma_{1}^{d}+\gamma_{2}^{d}}+\frac{\gamma_{1}^{p}\gamma_{2}^{p}}{\gamma_{1}^{p}+\gamma_{2}^{p}})$$

(4) $$\mathrm{Geometric}\;\mathrm{mean}:\;\gamma_{12}=\gamma_{1}+\gamma_{2}-2(\sqrt{\gamma_{1}^{d}\gamma_{2}^{d}}+\sqrt{\gamma_{1}^{p}\gamma_{2}^{p}})$$

In these equations, $\gamma_{1}$ is the surface energy of component 1, and $\gamma_{12}$ is the interfacial energy of components 1 and 2. Binding energy ($\omega_{12}$), has been widely used to predict the compatibility of two components and is defined as follows [36,37]:

(5) $$\omega_{12}=2(\sqrt{\gamma_{1}^{d}\gamma_{2}^{d}}+\sqrt{\gamma_{1}^{p}\gamma_{2}^{p}})$$

Atomic force microscopy topographic images (AFM, Veeco Instruments Inc., Santa Barbara, CA, USA) were obtained using a NanoScope IV MultiMode scanning probe microscope using the tapping mode probe with commercial Si_3_N_4_ integral tips. The topography observation was carried out at a constant amplitude of 40 mV and a resonance frequency of 463 kHz of the cantilever on 50 × 50 μm^2^ scan areas with a scan rate of 3 Hz. The samples were molded into rectangular strip with 2.5 × 2.5 cm^2^ and thickness of 0.5 mm.

A TECNAL 10 (Philips, Amsterdam, The Netherlands, accelerating voltage 120 KV) was used for transmission electron microscopy (TEM) analysis. The dispersion of hybrid GO/PEG in the PLA matrix was evaluated by TEM. TEM ultrathin sections were fabricated by a Powertome X (Boeckeler Instrument, Tucson, AZ, USA) cryo-ultramicrotome. Freshly sharpened diamond knives were used to obtain cryo-sections of 70–100 nm thickness flakes at an ambient temperature of −70 °C.

Scanning electron microscope (SEM) instrument (JSM-6390LV, JEOL Company, Tokyo, Japan) was used to investigate phase morphology. The GO/PEG composites with dimensions of 40 × 10 × 2 mm^3^ were fractured to expose the internal structure at room temperature. The GO/PEG/PLA specimens with dimensions of 40 × 10 × 2 mm^3^ were immersed in liquid nitrogen for approximately 15 min and fractured to expose the internal structure for SEM investigation. Before testing, all the specimens were gold sputtered to provide good conductivity.

A D8 ADVANCE (Bruker, Billerica, MA, America; Cu K_α_, λ = 0.154 nm, 40 kV, 40 mA) was used for wide angle X-ray diffraction (WAXD) analysis. The measurement was carried out at a 2θ angle of 3–80°, a scanning rate of 5°/min, and a scanning step of 0.02°.

Differential scanning calorimetry (DSC) was carried out using a Mettler Toledo DSC 1 Star System (Mettler Toledo, ZRH, Switzerland) equipped with a low-temperature accessory. The DSC measurements were performed from 25 to 210 °C at a heating rate of 10 °C/min, melted at 210 °C, and kept 3 min, then cooled to 10 °C at a cooling rate of 10 °C/min, kept at 10 °C for 3 min, and reheated to 210 °C at a heating rate of 10 °C/min for the second heating run. The glass transition temperatures (*T*_g_) of samples were taken from the midpoint of the stepwise specific heat increment. The degree of crystallinity (*X*_c_) for samples was determined according to the following Equation (6):

(6) $$X_{c}=\frac{\Delta H_{m}-\Delta H_{c}}{\omega \Delta H_{m}^{0}}\times 100\%$$

where Δ*H*_m_ and *ΔH*_c_ are the enthalpies of melting and cold crystallization, respectively. ω and $\Delta H_{m}^{0}$ are the weight fraction of PLA and melting enthalpy of 100% crystalline PLA, respectively. $\Delta H_{m}^{0}$ enthalpy of meting for 100% crystalline PLA (93.7 J/g) [39].

The crystallization morphology of different *M*_w_ of PEG, pure PLA, and a series of GO/PEG/PLA composites were obtained through a polarizing microscope (POM, Axioskop40, Zeiss, Oberkochen, Germany) with cooling-heating stage (BCS196, Linkam, Tadworth, UK). Small fragments of all samples were inserted between two microscope cover glasses and placed on a hot stage. The fragments were heated to 210 °C, kept at this temperature for 5 min, and then cooled to 10 °C.

Dynamic mechanical thermal properties of the PLA-based composites were investigated using a dynamic mechanical analysis (DMA 242, Netzsch, Bavaria, Germany). The three point bending method was used at a frequency of 1 Hz and a heating rate of 3 °C/min. The specimens were rectangular trips with dimensions of 60 × 10 × 4 mm^3^. The storage modulus (*E*′) and loss factor (tan*δ*) were measured in the case of temperature (10 to 120 °C).

Heat distortion temperature (HDT, Wallace Plastometer, Dorking, UK) under 0.455 MPa load of the various PLA-based composites was determined in accordance with ISO 75-2: 2003 method. The test pieces of 80 × 10 × 4 mm^3^ were heated at a rate of 2 °C/min in silicone oil bath, and the temperature when the test piece has deflected at 0.25 mm was reported as HDT value.

## 3. Results and Discussion

### 3.1. Contact Angle

**Figure 1:**
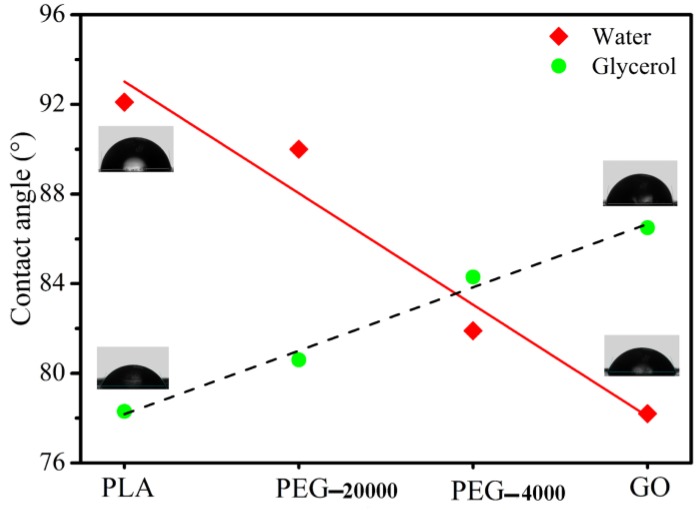
Photographs of contact angles (at 25 °C) and measured contact angles of sole sample.

As depicted in Figure 1, the water contact angle of the raw materials (from PLA, PEG-20000, PEG-4000 to GO) has a decreasing trend, while the corresponding glycerol contact angle has an increasing trend. The result indicates that the PLA has good hydrophobicity and the GO has good hydrophilicity. PLA with low polar groups concentration results in much more hydrophobicity as compared to GO. This behavior is originated from the structure of polymers since PLA has less concentration of hydrophilic groups (ester group) as compared to GO. Moreover, the PEG structure contains many hydrophilic hydroxyl groups making it more hydrophilic in comparison with PLA. Also, compared to equal amounts of PEG-20000, the PEG-4000 has more polar functional groups and shows a lower water contact angle, as represented in Figure 1. Deionized water and glycerol were selected as the test liquids for measuring contact angles. $\gamma^{d}$ for deionized water and glycerol was reported to be 21.8 and 34.0 mJ/m^2^, respectively, and $\gamma^{p}$ for the mentioned solvents was determined as 51.0 and 30.0 mJ/m^2^ in the same order [40]. Water and glycerol contact angles of PLA, PEG-20000, PEG-4000, and GO were measured 5 times and the mean of contact angles was listed in Table 2. Also, by knowing contact angles for each surface and using Equations (3) and (4), $\gamma^{d}$, $\gamma^{p}$ and γ of every component were listed in Table 2.

The calculated binding energy (ω) and interfacial energies of component couples based on harmonic and geometric mean equations are summarized in Table 3. The ω is always used to predict the compatibility between two components in polymer blends [36,37] and in the study, which are further calculated using the Equation (5). Notably, all of the ω for both samples existing in the PEG are larger than for those without PEG. The phenomenon suggests that the PEG with PLA and/or GO has good miscibility due to the fact that the PEG has a low *M*_w_ and a certain extent of amphiphilicity. Altogether, contact angle results indicated that the PEG is beneficial for enhanced compatibility of PLA-based composites in the melt-compounding process.

### 3.2. GO/PEG Morphology Analysis via SEM

**Figure 2:**
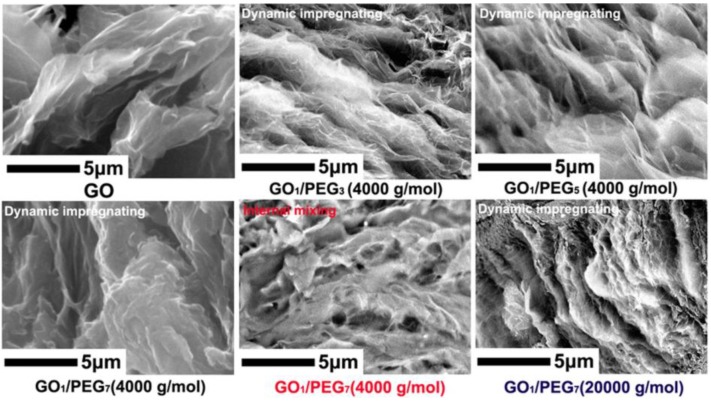
Scanning electron microscope (SEM) images of GO and the hybrid GO/PEG composites with different component ratio under different premixing method.

Figure 2 shows the SEM images of GO and hybrid GO/PEG composite with different component ratio and *M*_w_ of PEG under the different premixing method. As shown in Figure 2, it can be seen that GO exhibits a frizzy sheet shape and large amount of porous structure. Such porous structure remarkably increases its surface and the molten PEG can thus be adsorbed easily. For the hybrid GO/PEG composite with different component ratio under the dynamic impregnating, the fractured morphology of multiporous structure gradually disappears with the increase of PEG content. The phenomenon suggests that the PEG can be dispersed and adsorbed into the porous of GO via dynamic impregnating. PEG and GO are distinctly impregnated and covered by PEG. The capillary tubes and surface tension between PEG and GO can prevent the leakage of molten PEG [34]. Moreover, compared with dynamic impregnating, the fractured morphology of hybrid GO_1_/PEG_7_ composite based on the internal mixing shows an uneven flocculated structure. This phenomenon indicates that the molten PEG can insufficiently pour into the interlayers of GO. When the hybrid GO/PEG composite is with the *M*_w_ of PEG (20,000 g/mol), its fractured surface presents clearly a flocculated structure and agglomeration; the result might be due to the inferior mobility of high *M*_w_ of PEG at given temperature.

### 3.3. Composites Morphology Analysis via AFM

**Figure 3:**
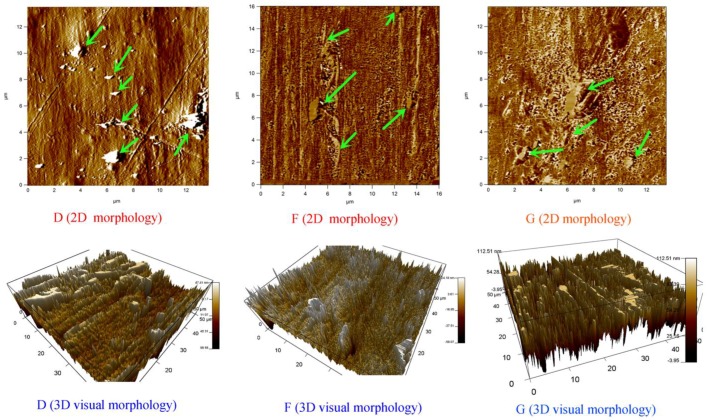
2D grayscale morphologies of (D) GO_1_/PEG_3_/PLA_96_ and (F) GO_1_/PEG_7_/PLA_92_ based on dynamic impregnating, (G) GO_1_/PEG_7_/PLA_92_ based on internal mixing and corresponding 3D visual morphologies obtained using AFM.

The AFM schemes obviously showed that the samples exhibit typical ‘sea-island’ morphologies, where discrete flake shape domains consist of GO dispersed in the PLA matrix. To better visualize the morphologies of these blends, the AFM grayscale 2D up-view of GO_1_/PEG_3_/PLA_96_ (D) and GO_1_/PEG_7_/PLA_92_ (F) via dynamic impregnating, and GO_1_/PEG_7_/PLA_92_ (G) via internal mixing, are also depicted in the Figure 3. These 2D schemes clearly show the contrast of the PLA and GO phases. In these ternary blends, the attached PEG to GO could act as a dispersing agent for the GO/PEG phase, and thus, the hybrid GO/PEG granules can efficiently flow in the melt state similar to thermoplastic materials. Thus, the hybrid GO/PEG via dynamic impregnating has better dispersity and absorbability than that of internal mixing, leading to the smaller size of GO in the PLA matrix.

The AFM micrographs of GO_1_/PEG_3_/PLA_96_ (D) and GO_1_/PEG_7_/PLA_92_ (F) via dynamic impregnating, and GO_1_/PEG_7_/PLA_92_ (G) via internal mixing, are shown in the 3D images of Figure 3, respectively. As depicted in Figure 3, there can be found flake GO particles distributed in the PLA matrix. This sea-island morphology is stable even by changing the concentration of PEG, but the size of GO particles becomes smaller when PEG concentration increases to 7 wt %. Moreover, when the PEG weight concentration is 7 wt %, compared with the internal mixing the size of GO particles in the PLA matrix is smaller via dynamic impregnating.

### 3.4. Composites Morphology Analysis via TEM

**Figure 4:**
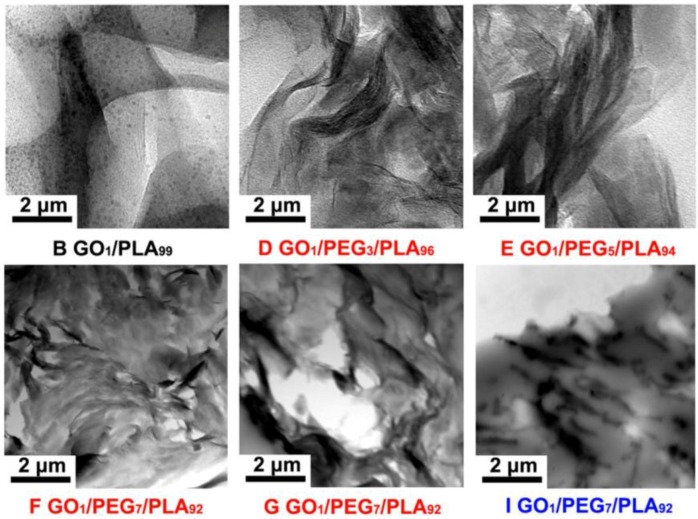
Transmission electron microscopy (TEM) images of GO/PLA and GO/PEG/PLA multi-phase composites with the introduction of GO/PEG of different component ratios under different melt-compounding.

TEM observation can clearly distinguish the inner phase morphology and nanosized dispersed phase of multiphase materials. The dispersion morphology of hybrid GO/PEG particles in the PLA matrix in the case of different component ratios, premixing method, and *M*_w_ of PEG are shown in Figure 4, respectively. The dark thread-like and irregular scale objects dispersed in the PLA matrix are identified as GO platelets, as shown in Figure 4. Interestingly, the dispersed morphology of GO particles becomes fuzzier with the increasing of PEG (4000 g/mol) content, as shown in Figure 4D–F. The phenomenon implies that the hybrid GO/PEG with high concentration of PEG has formed well exfoliated morphology in the PLA matrix. Compared to the hybrid GO/PEG via different premixing method, as shown in Figure 4F,G, and compared with dynamic impregnating, a spot of flocculated GO particles emerges in the PLA matrix under the internal mixing. It can be due to the excellent dispersibility and adsorbability between GO and PEG based on the dynamic impregnating. In addition, when *M*_w_ of the PEG is 20,000 g/mol, the hybrid GO/PEG particles of the PLA-based composites show evidently flocculated and/or agglomerated morphologies, as shown in Figure 4I. The result suggests that the PEG (20,000 g/mol) cannot be availably infused into the interlamination of GO particles to form uniform exfoliated morphology in the PLA matrix.

### 3.5. Composites Morphology Analysis via SEM

**Figure 5:**
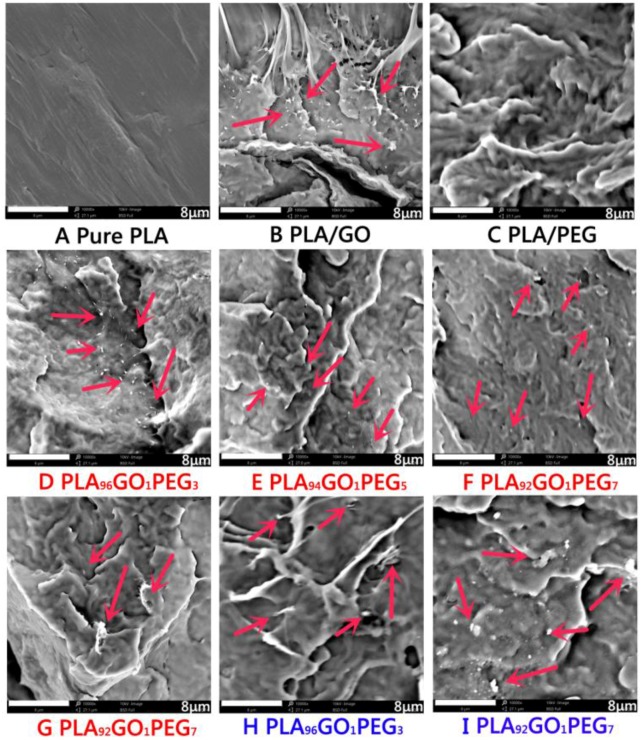
SEM images of pure PLA, GO/PLA, PEG/PLA, and GO/PEG/PLA multi-phase composites with different component ratio under different melt-compounding.

Figure 5 shows the SEM images of pure PLA, GO/PLA, PEG/PLA, and a series of GO/PEG/PLA composites with different premixed hybrid GO/PEG. For the GO/PLA composites (1 wt % of GO), the dispersed morphology of the short fibers and droplets is shown in Figure 5B. The morphology of undulated fracture surface with the introduction of PEG was clearly exhibited, as shown in Figure 5C. The result suggests that the low *M*_w_ of PEG contributed to enhancing the plasticity of PLA. Moreover, compared with GO/PLA composite, the disperse morphology of GO/PEG/PLA composites with the hybrid GO/PEG based on dynamic impregnating shows more fine and uniform flakes in the PLA matrix, as shown in Figure 5D–F. Also, the hybrid GO/PEG via dynamic impregnating exhibits more fine particles in the PLA matrix than that of internal mixing (as depicted in Figure 5G). On the one hand, these results demonstrate that the dispersibility of hybrid GO/PEG in the PLA can be improved using low *M*_w_ of PEG. On the other hand, the dynamic impregnating device can generate strong synergy effects of supersonic vibrating and vacuuming force field, which enhance the dispersed morphology of hybrid GO/PEG in the PLA matrix during the melt compounding process.

Furthermore, when the hybrid GO/PEG with the *M*_w_ of 20,000 g/mol under dynamic impregnating was introduced into the PLA matrix, the dispersed morphology of flocculated flakes was exhibited in the PLA, as depicted in Figure 5H,I. Compared with the GO/PEG/PLA composites (*M*_w_ of PEG: 4000 g/mol) via dynamic impregnating, dispersion particle size of the GO/PEG/PLA composites (*M*_w_ of PEG: 20,000 g/mol) was distinctly increased. The phenomenon suggests that the high *M*_w_ of PEG poured into the lamellas of GO with more difficultly than that of low *M*_w_ under melt-compounding.

### 3.6. Composites Crystallization Behavior via WAXD

**Figure 6:**
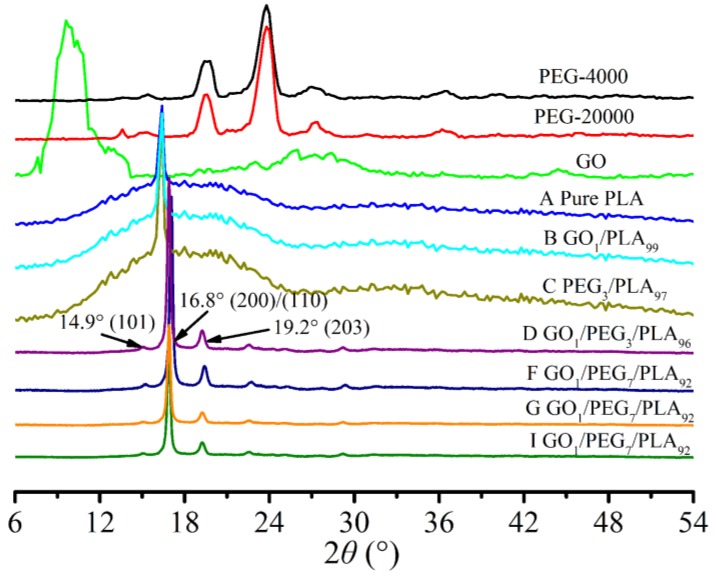
Wide angle X-ray diffraction (WAXD) diffraction patterns of PEG, pure PLA, GO/PLA, PEG/PLA, and GO/PEG/PLA multi-phase composites with different component ratio under different melt-compounding.

Figure 6 presents the WXRD patterns of PEG, GO, pure PLA, GO/PLA, PEG/PLA, and GO/PEG/PLA composites with various PEG weight percentages under different premixing methods. As shown in Figure 6, the particular diffraction peak located at nearby 10.1° for GO indicates that graphite stack is separated into various amounts of regular flakes by the Hummers method. Either *M*_w_ of 4000 or 20,000 g/mol, and the strong diffraction peaks located at approximately 19.6° and 24.0° for PEG demonstrate that PEG is typical crystalline polymer. After mixing the GO and/or PEG with PLA, the peak corresponding to crystal space of GO and/or PEG was not clearly observed. This result implies that some exfoliation of GO in the polymer matrix has occurred and PEG cannot form perfect crystal within the GO and PLA. This structure might contribute to the storage modulus and heat distortion temperature of the GO/PEG/PLA nanocomposite.

Moreover, the evident diffraction shoulder located at nearby 16.8° for pure PLA may be owed to low crystallinity of the PLA and its imperfect crystal. Interestingly, when the hybrid GO/PEG was introduced into the PLA, the strong diffraction peaks located at approximately 14.9°, 16.8° and 19.2° have appeared; these positions of the diffraction peaks in the X-ray scan for the PLA-based composites with different component ratio, *M*_w_ of PEG, and premixing method are insignificantly different. This result means that the introduction of the hybrid GO/PEG can effectively improve the crystal structure of PEG. Also, this result suggests that the amorphous GO acts as a nucleating agent and PEG acts as a plasticizer in PLA matrix, and can accelerate the nucleation and its crystal growth of PLA.

### 3.7. Composites Crystallization Behavior via DSC

**Figure 7:**
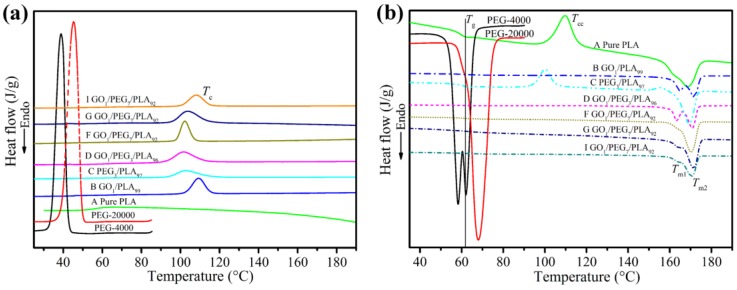
Differential scanning calorimetry (DSC) curves of PEG, pure PLA, GO/PLA, PEG/PLA, and GO/PEG/PLA multi-phase composites with different component ratio under different melt-compounding. (**a**) Cooling curves; (**b**) melting curves.

To investigate the thermal behavior of PLA-based composites, DSC measurements were carried out. DSC curves (cooling and melting process) of PEG, pure PLA, GO/PLA, PEG/PLA, and a series of GO/PEG/PLA composites are presented in Figure 7. Thermal properties of these materials, such as glass transition temperature (*T*_g_), crystallization temperature (*T*_c_), cooling crystallization temperature (*T*_cc_), melting temperature (*T*_m_), crystallization enthalpy ($\Delta H_{c}$) melting enthalpy ($\Delta H_{m}$), and crystallinity ($X_{c}$), are reported in Table 4.

As depicted as in Figure 7a, the intense crystallization peaks for PEG of 4000 and 20,000 g/mol locate on 38 and 44 °C, respectively, while PLA is not obvious crystallization peak in the temperature rang. The phenomenon further indicates that the PEG is a typical crystal polymer and the PLA is a semicrystalline polymer with slow rate of crystallization. However, the addition of GO and/or PEG to PLA increases crystallization peak due to the enhanced nucleation points and/or mobility of PLA chains [22]. Moreover, synergy effect of GO and PEG further strengthens the crystallization peak of the PLA. The melting behaviors of PEG, pure PLA, GO/PLA, PEG/PLA, and a series of GO/PEG/PLA composites are presented in the Figure 7b. It can see the strong melting peaks located on nearly 60 and 70 °C for PEG (4000 and 20,000 g/mol), respectively. Also, pure PLA exhibits the clear *T*_g_ (nearly 62 °C), *T*_cc_ (nearly 108 °C), and *T*_m_ (nearly 165 °C). Notably, the cooling crystallization behavior of PLA disappears and weakens with the introduction of GO and PEG, respectively. The results clearly demonstrate that GO and PEG can act a high-efficiency nucleating agent and plasticizer. Compared with pure PLA, all of *T*_m_ of the PLA-based composites have slightly increase with the addition of hybrid GO/PEG. The phenomenon indicates that not only can the hybrid GO/PEG can improve crystallization but it can also hinder the motion of PLA chain in melting process.

In addition, thermal properties of PLA-based composites as a function of the GO/PEG with different component ratio and premixing method are shown in Table 4. As is well-known, due to the slow rate of crystallization in PLA, its chains had not enough time to form ordered chains [22,25]. We can see that the *T*_cc_ of PLA phase in the composites disappears by the addition of GO, which is because of the high efficiency nucleating effect of this material for the PLA phase. Also, the $\Delta H_{\mathrm{cc}}$ of PLA phase in the PEG/PLA blend is decreased by the addition of PEG; the result implies that the PEG plasticization effect causes the higher flow-ability of PLA chains during melt-blending, inducing a higher level of crystallization. Moreover, when the PEG has adopted *M*_w_ of 4000 g/mol, compared with the GO_1_/PEG_3_/PLA_96_ composite (as shown in Table 4D), the $\Delta H_{m}$ and $X_{c}$ of GO_1_/PEG_7_/PLA_92_ composite (as shown in Table 4F) increase by 6.3 J/g and 6.7%, respectively. The phenomenon suggests that the addition of more PEG causes a better dispersed morphology of GO and a higher level of PLA chains motion in the produced composites, which causes a quicker rate of crystallization of the PLA phase. The increase of crystallinity when PEG was the dispersed phase within the PLA matrix was also reported in previous studies [34,35], since the interface of such system can serve as a plasticizer during the crystallization stage of PLA [22]. However, the *M*_w_ of PEG was increased by 20,000 g/mol, and the $\Delta H_{m}$ and $X_{c}$ of PLA phase in the GO/PEG/PLA composite were, to a certain extent, decreased. Altogether, the synergy effect of the hybrid GO/PEG can significantly improve rate of crystallization of the PLA phase during the melt-compounding process. Therefore, with the higher *T*_m_, $\Delta H_{c}$, $\Delta H_{m}$ and *X*_c_ of the GO/PEG/PLA composites being comparable to pure PLA, the mentioned parameters in the composites can be attributed to the enhanced nucleating points and high rate of crystallization of the PLA using the GO and PEG.

### 3.8. Composites Crystallization Behavior via POM

**Figure 8:**
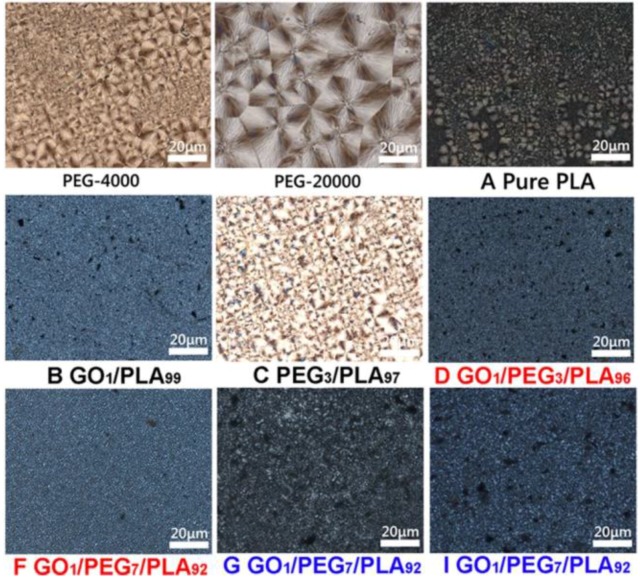
Polarizing microscope (POM) images of PEG, pure PLA, GO/PLA, PEG/PLA, and GO/PEG/PLA multi-phase composites with different component ratio under isothermal crystallization.

The POM photographs of PEG, pure PLA, GO/PLA, PEG/PLA, and a series of GO/PEG/PLA composites are shown in Figure 8. The spherulites morphology of pure PEG (either 4000 or 20,000 g/mol) is fine and close under melting/cooling process. Compared to pure PEG, the spherulites morphology of pure PLA is of considerable faultiness during crystal process, as shown in Figure 8A. The phenomenon is due to the intrinsic slow rate of crystallization of PLA. Also, the spherulites morphology of GO/PLA composite (as shown in Figure 8B) is smaller, and more faulty at GO content of 1 wt % compared with that of pure PLA. The result implies that GO may be a nucleating agent for PLA crystallization. As depicted in Figure 8C, the finer and more spherulite part of the PLA phase with the 3 wt % of PEG is exhibited. The result suggests that PEG can be an efficiency plasticizer to accelerate the motion of PLA molecular chains. Moreover, compared to the hybrid GO/PEG with low *M*_w_ of PEG (as shown in Figure 8D–F), POM photographs of the GO/PEG/PLA composites (as shown in Figure 8G–I) as a function of high *M*_w_ of PEG have more black flakes. The result should contribute to the inferior dispersibility of hybrid GO/PEG within the PLA phase.

### 3.9. Composites Thermal Behavior via DMA

**Figure 9:**
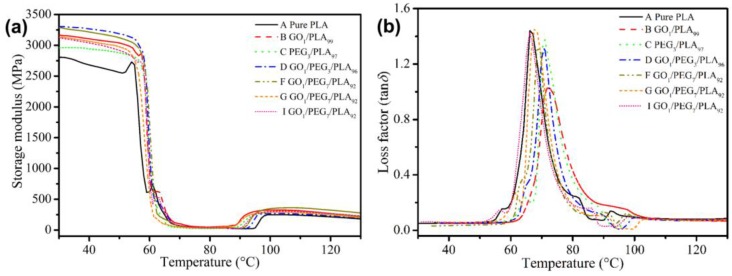
DMA curves of pure PLA, GO/PLA, PEG/PLA, and GO/PEG/PLA multi-phase composites in the case of different component ratio. (**a**) Storage modulus curves; (**b**) loss factor curves.

Dynamic mechanical analysis (DMA) was performed to study chain motion at the glassy/rubbery state of the polymer. Figure 9a exhibits the storage modulus (*E*′) of pure PLA, GO/PLA, PEG/PLA, and a series of GO/PEG/PLA composites. Compared with pure PLA, *E*′ of the PLA-based composite with the addition of GO or PEG are increased, respectively. On the one hand, the result indicates that the GO addition could effectively enhance stiffness of PLA matrix. On the other hand, the increased *E*′ of the PEG/PLA composite is due to the high crystallinity of PLA phase induced by PEG. Moreover, compared with GO/PLA composite, *E*′ of the PEG/PLA composites slightly decreased, ranging from 30 to 130 °C. The result indicates that a small amount of uncombined PEG located in the amorphous region of PLA phase leads to decrease of stiffness of PLA matrix. The *E*′ of the GO/PEG/PLA composites decreased with the increase of PEG content. The result is contributed to the external plasticization of PEG in the PLA matrix, resulting in the decrease of storage modulus. Importantly, compared with the GO/PEG/PLA composites with low *M*_w_ of PEG (4000 g/mol), *E*′ of the GO/PEG/PLA composites with high *M*_w_ of PEG (20,000 g/mol) showed a certain extent of decline at a specific temperature range (30–60 °C), indicating the decrease of strength of the composites. This is due to flocculated and/or agglomerated particles of the hybrid GO/PEG with high *M*_w_ of PEG. Owing to the synergy effect of GO and PEG, *E*′ of the composites with hybrid GO/PEG via dynamic impregnating was higher than that of PLA ranging from 30 to 130 °C. The phenomenon implies that the hybrid GO/PEG can effectively enhance strength of PLA, which produces a broader industrial application.

The loss factor (tan δ) of the pure PLA, GO/PLA, PEG/PLA, and a series of GO/PEG/PLA composites is shown in Figure 9b. Compared with the *T*_g_ (nearby 68 °C) of pure PLA, the GO/PLA composite with 1 wt % of GO shows a slight increase. THis could be due to the high crystallinity of PLA phase and the inhibition effect of PLA chains as a function of GO. Moreover, either low *M*_w_ or high *M*_w_ of PEG, and *T*_g_ of the composites, show a slight increase with the introduction of PEG and/or hybrid GO/PEG. The phenomenon clearly indicates that the PEG not only evidently enhances the dispersibility of GO in PLA matrix but also improves the crystallinity of PLA phase. This result is in agreement with the aforementioned analysis, including the SEM and DSC observation.

### 3.10. Composites Thermal Behavior via HDT

**Figure 10:**
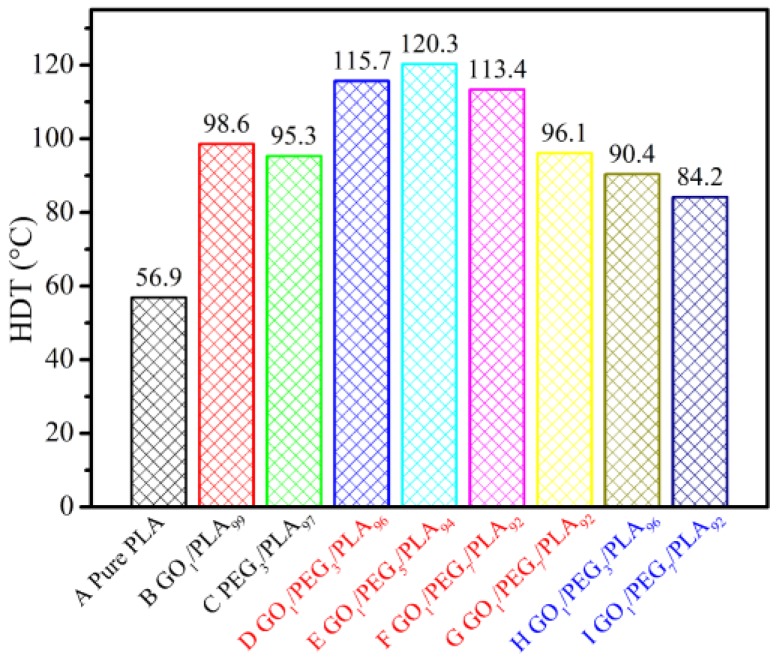
HDT of pure PLA, GO/PLA, PEG/PLA, and GO/PEG/PLA multi-phase composites in the case of different component ratio.

Figure 10 shows the HDT value of pure PLA, GO/PLA, PEG/PLA, and a series of GO/PEG/PLA specimens. From the heat distortion temperature (HDT) results, it seems that the PLA composite containing the hybrid GO_1_/PEG_5_ is the best formulation (at 120 °C), provided that the fabricated hybrid GO/PEG with low *M*_w_ of PEG (4000 g/mol) was premixed by dynamic impregnating. Therefore, further attempts were made to investigate the synergy effect of GO and PEG on thermomechanical properties of the composite. Moreover, for the GO_1_/PEG_7_/PLA_92_ composite, the HDT via dynamic impregnating is higher than that of internal mixing (approximately 17 °C). The result indicates that the synergy effect of supersonic vibrating and vacuuming force field can markedly enhance dispersed and adsorbed morphology between GO and PEG, leading to excellent dispersion of the hybrid GO/PEG within the PLA matrix. Furthermore, change in HDT value was followed by *M*_w_ of PEG. When the *M*_w_ of PEG is 20,000 g/mol, HDT for the GO/PEG/PLA composites evidently decreases. These results are in good agreement with the isothermal DSC thermograms of various PLA-based composites (Figure 7). On the other hand, by mixing with the hybrid GO/PEG, the isothermal crystallization of PLA was completed. Percentage crystallinity of the PLA-based composites with the hybrid GO/PEG calculated from the isothermal DSC thermogram is significantly higher than that of pure PLA.

### 3.11. Physical Model for Morphology and Crystallization

**Figure 11:**
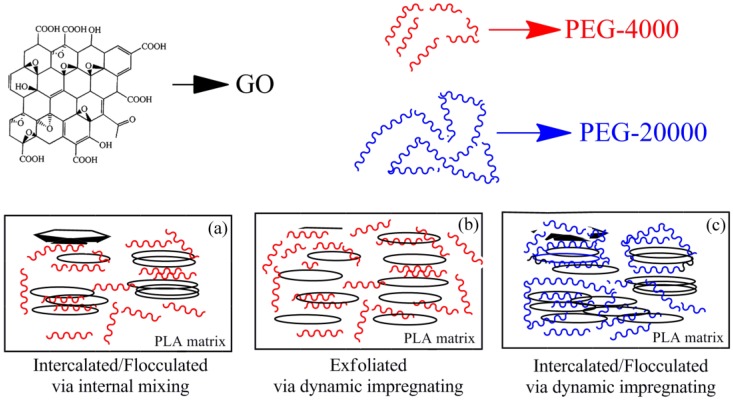
Physical model for dispersed morphologies of GO/PEG in PLA matrix with different molecular weight via dynamic impregnating and/or internal mixing, respectively. (**a**) represents GO/PEG-4000/PLA nanocomposite via internal mixing, (**b**) represents GO/PEG-4000/PLA nanocomposite via dynamic impregnating and (**c**) represents GO/PEG-20000/PLA nanocomposite via dynamic impregnating.

A significant enhancement in the crystallinity, storage modulus, and HDT of the PLA phase were observed for a uniform exfoliated structure (as depicted in Figure 11b) when the hybrid GO/PEG with different component ratio under dynamic impregnating was used for PLA-based nanocomposite preparations (either the hybrid GO/PEG (*M*_w_ of PEG: 4000 g/mol) via the internal mixing or the hybrid GO/PEG (*M*_w_ of PEG: 20,000 g/mol) under the dynamic impregnating), which gave a certain amount of intercalated/flocculated structure (as depicted in Figure 11a or Figure 11c) with upper HDT values compared to that of the PLA. Due to the strong interaction between hydroxylated, carboxylation, and epoxide edge–edge groups, the GO particles are sometimes flocculated in the polymer matrix. As a result of this flocculation, the length of the GO particles increases slightly, resulting in a corresponding increase in overall aspect ratio. On the other hand, the flocculated structure of GO/PEG/PLA composites also can lead to the agglomeration of GO within the PLA phase. For this reason, the flocculated structure of dispersed GO particles is much higher in the case of GO/PEG/PLA composites (as depicted in Figure 11a or Figure 11c) compared to the other nanocomposites (as depicted in Figure 11b), hence the low enhancement of HDT values. Depending on the synergy effect of supersonic vibrating and vacuuming force field, excellent dispersed and adsorbed structure of the hybrid GO/PEG is achievable, which can improve the crystallization rate of the PLA and enhance the HDT value.

## 4. Conclusions

This study presented that the low crystallinity and HDT value of PLA could be improved by the addition of the highly dispersed hybrid GO/PEG, and thereby, the resulted crystallinity could be enhanced from 5.5 to 39.3% and the HDT value changed from 56.9 to 120.3 °C by incorporating the hybrid GO/PEG via the dynamic impregnating method. In this work, a novel premixing method was introduced to the system for improving dispersibility and adsorbability between GO and PEG, which also caused the more exfoliated structure of the hybrid GO/PEG within the PLA matrix during the melt-compounding process and further enhanced the crystallization rate of the PLA phase.

This dispersed morphology was confirmed by SEM, AFM, and TEM observations. Compared with the internal mixing premixing method, the hybrid GO/PEG within the PLA matrix exhibited a well exfoliated morphology in which the size of the dispersed GO particles became smaller with 5 wt % of PEG under the dynamic impregnating premixing method. The crystallization behaviors of the PLA-based composites were studied by WAXD, DSC, and POM measurements. When the *M*_w_ of PEG adopted 4000 g/mol with the introduction of hybrid GO/PEG via the dynamic impregnating and/or internal mixing, *X*_c_ of the PLA phase was significantly improved, which led to the increase of the HDT value for the composites. While the *M*_w_ of PEG was 20,000 g/mol, *X*_c_ of the PLA-based composites with the hybrid GO/PEG was also to a certain degree of improved compared to the pure PLA. DMA and HDT results indicated that the exfoliated morphology of the hybrid GO/PEG within the PLA matrix can more effectively increase the storage modulus and HDT value of the PLA-based composite. The reported results reveal that the composites, especially the GO/PEG/PLA composite with the hybrid GO/PEG (low *M*_w_ of PEG) under the dynamic impregnating premixing method, along with their crystallization rate, crystallinity, storage modulus, and HDT value, are fully desirable which can be used in a wide range of industry applications.

## Figures and Tables

**Table 1:** Different formulations of poly-(lactic acid) (PLA)-based composites with the introduction of graphene oxide (GO)/polyethylene glycol (PEG).

Sample	GO (wt %)	PEG (wt %)	PLA (wt %)	*M*_w_ of PEG (g/mol)	Premixing Method for Hybrid GO/PEG
A	---	---	100	---	---
B	1.0	---	99	---	---
C	---	3.0	97	4000	---
D	1.0	3.0	96	4000	Dynamic impregnating
E	1.0	5.0	94	4000	Dynamic impregnating
F	1.0	7.0	92	4000	Dynamic impregnating
G	1.0	7.0	92	4000	Internal mixing
H	1.0	3.0	96	20,000	Dynamic impregnating
I	1.0	7.0	92	20,000	Dynamic impregnating

**Table 2:** The contact angles (25 °C) and surface energies of sample components.

Sample	Contact Angle (°)		Surface Energy (mJ/m^2^)		
	Water	Glycerol	γ	$\gamma^{d}$	$\gamma^{p}$
Water	---	---	72.8	21.8	51.0
Glycerol	---	---	64.0	34.0	30.0
PLA	92.1	77.3	27.8	26.1	2.8
PEG-_20000_	90	80.6	23.3	18.1	5.2
PEG-_4000_	81.9	84.3	28.0	2.2	25.8
GO	78.2	86.5	34.6	0.4	34.2

**Table 3:** The values of component couple surface energies and binding energies of the samples.

Component Couple	$\gamma_{12}^{a}$ (mJ·m^−2^)	$\gamma_{12}^{b}$ (mJ·m^−2^)	ω (mJ·m^−2^)
PLA/GO	50.5	36.4	25.0
PLA/PEG-_4000_	37.6	23.6	32.2
PLA/PEG-_20000_	34.7	26.2	27.9
GO/PEG-_4000_	2.4	1.3	61.3
GO/PEG-_20000_	38.3	57.9	32.1

^a^: via the harmonic mean equation. ^b^: via the geometricmean equation.

**Table 4:** Thermal properties of PLA-based composites as a function of different GO/PEG.

Samples	$T_{m1}$ (°C)	$T_{m2}$ (°C)	$T_{\mathrm{cc}}$ (°C)	$T_{c}$ (°C)	$\Delta H_{m}$ (J/g)	$\Delta H_{\mathrm{cc}}$ (J/g)	$\Delta H_{c}$ (J/g)	$X_{c}$ (%)
PEG-_4000_	58.2	61.9	---	39.1	154.1	---	149.6	72.3
PEG-_20,000_	---	68.3	---	44.8	157.3	---	153.5	73.9
A	161.3	168.5	110.1	---	33.7	28.5	9.78	5.5
B	164.7	171.6	---	99.6	29.8	---	26.3	31.8
C	---	169.4	100.3	103.2	34.1	15.9	14.6	19.4
D	163.8	170.1	---	102.7	30.5	---	15.3	32.6
F	164.2	170.2	---	102.9	36.8	---	24.3	39.3
G	163.9	172.7	---	104.4	28.7	---	13.5	30.6
I	163.1	170.3	---	108.3	25.4	---	11.8	27.1

*T*_m1_, *T*_m2_, and *T*_cc_ represent melting temperature 1, melting temperature 2, and cooling crystallization temperature of second DSC scan, respectively. The melting enthalpy of 100% crystalline PEG and PLA employ 93.7 J/g [39] and 213.0 J/g [41].
